# Berberine-10-hydroxy camptothecine-loaded lipid microsphere for the synergistic treatment of liver cancer by inhibiting topoisomerase and HIF-1α

**DOI:** 10.1080/10717544.2020.1870020

**Published:** 2021-01-11

**Authors:** Yingjie Qi, Guangxuan Liu

**Affiliations:** Department of Pharmacy, Cancer Hospital of China Medical University, Liaoning Cancer Hospital & Institute, Shenyang, P. R. China

**Keywords:** BBR, 10-HCPT, lipid microsphere, liver cancer, synergistic treatment

## Abstract

10-HCPT is a topoisomerase I inhibitor effective in the treatment of liver cancer but its use is hampered by its resistance. The expression of hypoxia-inducible factor-1α (HIF-1α) is reportedly upregulated in liver cancer tissues, which is directly linked to the resistance of 10-HCPT. While BBR can significantly decrease the level of HIF-1α according to the literature report. Thus, the aim of this study was to prepare a novel intravenous 10-HCPT-BBR-loaded lipid microsphere (LM) and evaluate their synergistic effect on liver cancer treatment. The optimal preparation mainly included 10.0% oil phase (medium-chain triglyceride:long-chain triglyceride = 1:1), emulsifier (egg lecithin E80 and pluronic F68), antioxidant (0.02% NaHSO_3_), and pH regulator (0.1 mol/L Hcl). Then, the behaviors of BBR-10-HCPT loaded LM *in vitro* and *in vivo* were systematically investigated. *In vitro*, it showed an obvious sustained-release effect in different release mediums, good physicochemical stability at accelerated and long-term storage conditions, and great anti-proliferative capability toward human liver cancer Hep-3B cells. *In vivo*, the prepared LM exhibited a longer half-life and higher AUC compared to BBR injection and 10-HCPT injection. More importantly, it was found that The LM was distributed more in the liver, spleen, and tumors, but less in the lungs and heart, especially in the lung. And then, it showed significant inhibition of tumor growth against nude mouse with Hep-3B tumor, and the tumor inhibition rate reached 91.55%. Thus, the data obtained in our study suggested that BBR combined with 10-HCPT can raise curative effect and reduce the toxicity of 10-HCPT.

## 1. Introduction

Liver cancer is the second leading cause of cancer-related deaths in the world, with more than 50.0% of the total number of cases and deaths occur in China (Fu & Wang, 2018; An et al., 2019). Liver cancer has clinical characteristics such as insidious onset, rapid progress, early recurrence, and poor prognosis (Jacobs, 2015; Forner et al., 2018; Alanazi et al., 2020). Most of it has reached the advanced stage when clinically discovered, and patients have lost the opportunity for surgical treatment. Therefore, chemotherapy, especially small molecule cytotoxic drugs-based chemotherapy, is still an important strategy for the treatment of advanced liver cancer. However, its application is limited due to lack of selectivity for tumor cells over normal cells resulting in insufficient drug concentrations in tumors, systemic toxicity, and the appearance of drug-resistant tumor cells (Chu & Dupuy, 2014; Knox et al., 2015; Li, Yin, et al., 2019). Therefore, it is necessary to develop new anti-tumor drugs with high efficiency, low toxicity, and multi-target for the treatment of liver cancer.

HCPT is one of the camptothecin (CPT) analogs with a powerful cytotoxic effect and a broad range of anticancer activity for many cancers. The molecular target of the CPTs is the topo I, which is over-expressed in lots of tumor cell lines and involve in the regularization of DNA topology (Li et al., 2016). However, it was noticed that although 10-HCPT showed a strong antitumor effect on liver cancer cells *in vitro*, it’s *in vivo* antitumor efficacy was limited, which may be related to the microenvironment of liver cancer. It is well known that the blood supply is limited in liver cancer, especially for liver cancer with cirrhosis, which ultimately leads to local hypoxia. While the high expression of HIF-1α induced by hypoxia was highly associated with the resistance of topoisomerase I (topo I) inhibitors, such as 10-HCPT (Dubbelboer et al., 2019; Govaert et al., 2014). Berberine hydrochloride (BBR), a class of drugs that regulate the intestinal flora, shows strong activity against a variety of tumors (Pan et al., 2017; Huang et al., 2018; Li et al., 2019), including liver cancer, ovarian cancer, lung cancer, colon cancer, breast cancer, by inhibiting topoisomerase I/II, NF-κB activity, and its pathway, decreasing the level of HIF-1α and inducing apoptosis (especially the up-regulation of Fas-mediated apoptosis and the inhibition of the arachidonic acid metabolic pathway and the PI3K/AKT pathway) (Wang et al., 2009; Yip & Ho, 2013; Li et al., 2015; Chu et al., 2016; Li et al., 2017; Song et al., 2019). Thus, the combination of 10-HCPT and BBR could produce a synergistic effect in antitumor activity because BBR could increase the antitumor activity of 10-HCPT by decreasing the level of HIF-1α. It is well known that nano-drug delivery systems can not only improve the bioavailability of drugs but also significantly decrease drug toxicity (Wang et al., 2011; Zhang et al., 2014; Mirhadi et al., 2018). So how to construct a novel drug delivery system suitable for loading and delivering BBR and 10-HCPT has great research value because of the drawbacks of BBR and 10-HCPT, such as poor oral bioavailability, low aqueous solubility, and severe toxicity of intravenous injection (Cui et al., 2018; Elsheikh et al., 2018).

Among nano-delivery systems, the lipid microsphere (LM) has drawn great attention in recent years because of its unique properties, (Teng et al., 2014; Luo et al., 2017, 2018) including: (1) The lipids spatial structure of LM allows greater drug loading and better stability compared to other nanomedicines, such as liposomes, nanoparticles, and micelles; (2) LM has high injection safety and low irritation because LM can tolerate thermal terminal sterilization. But other nanomedicines usually use the strict aseptic operation to ensure sterility; (3) Lecithin-based LM possesses excellent biocompatibility because lecithin mixtures as the main emulsifiers are a component of biofilms. Hence LM has natural advantages as a delivery system; (4) LM has good passive targeting ability toward tumors based on the EPR effect because LM possesses controlled particle size, which will contribute to a more selective distribution of drugs at tumor tissue; (5) LM can protect drugs in oil from biodegradation and prevent drugs from coming into direct contact with the blood to extend *in vivo* circulation time. Hence, LM is a prospective nano-delivery system for solving the problems faced by antitumor drugs like BBR and 10-HCPT. Additionally, compared to those reported nano-delivery systems, the preparation process is highly efficient and well controllable, with high potential for possible industrial automation and massive production.

In this paper, a novel BBR-10-HCPT-loaded LM was developed and prepared by high-pressure homogenization method to treat liver cancer. *In vitro,* the particle size distribution, drug release behavior (in phosphate buffer and plasma diluent), factors affecting LM physicochemical stability (sterilization conditions, pH values, oil phase, emulsifier), and the cytotoxicity of BBR-10-HCPT-loaded LM were studied systematically. Meanwhile, the pharmacokinetics, pharmacodynamics, and tissue distribution behavior in rat or nude mouse models were investigated in order to evaluate BBR-10-HCPT-loaded LM’s *in vivo* behavior accurately. It was found that BBR-10-HCPT-loaded LM exhibited longer *t*_1/2_, larger AUC, smaller CL, and higher liver and tumor tissue accumulation compared with injections. To sum up, BBR-10-HCPT-loaded LM might be a promising nanomedicine for the treatment of liver cancer.

## 2. Materials and methods

### 2.1. Materials

10-HCPT and 10-HCPT injection (1.0 mg/mL) were purchased from Wuhan Lishizhen Pharmaceutical Co., Ltd. (Wuhan, China). BBR and tetrahydropalmatine were purchased from Northeast Pharmaceutical Group Co., Ltd. (Shenyang, China) and the National Institute for the Control of Pharmaceutical and Biological Products (Beijing, China) separately. Egg lecithin (S75, E80) and oleic acid were obtained from Lipoid GmbH (Shanghai, China). Medium-chain triglyceride (MCT) and long-chain triglyceride (LCT) were obtained from TieLing BeiYa Pharmaceutical Corporation. Glycerin and pluronic F68 were purchased from Shanghai Xin Fan Biological Technology Co. Ltd. (Shanghai, China). Dehydrated alcohol, acetonitrile and methanol were purchased from Fisher Scientific. Purified water was obtained using a Milli-Q^®^ ultrapure water system (USA). Hep-3B cells were purchased from Shanghai Honsun Biological Technology Co., Ltd (Shanghai, China). Nude mice and SD rats were purchased from Beijing HFK Bioscience Co. Ltd (Beijing, China). Rat enzyme-linked immunosorbent assay (ELISA) kit was purchased from ZhiWei Biology Co. Ltd (Hefei, China).

### 2.2. Preparation of BBR-10-HCPT-loaded LM

BBR-10-HCPT-loaded LM was prepared by the high-pressure homogenization method, and the concentration of BBR and 10-HCPT was 3.0 mg/mL and 1.0 mg/mL respectively. The detailed preparation processes were as follows: 3.5% (w/v) E80, 0.3% (w/v) BBR, and 0.1% (w/v) 10-HCPT were dissolved in dehydrated alcohol (5.0%, v/v). After the mixture was completely dissolved, the resulting solutions were evaporated under nitrogen flow. Then, adding oleic acid (0.025%, w/v), LCT (5.0%, w/v), and MCT (5.0%, w/v) to the phospholipid complex, and the mixture was heated with constant stirring at 70.0 °C until it became a transparent solution. 2.0% (w/v) glycerin, 0.02% (w/v) sodium bisulfite and 0.25% (w/v) pluronic F68 were dissolved in water at 70.0 °C to acquire the aqueous phase. The coarse emulsion would be obtained by adding the oil phase to the aqueous phase with continuous stirring of high-speed shear mixing at 15,000 rpm for 2.0 min after the oil and water phases were completely dissolved. Then, the volume of the coarse emulsion was replenished to 100% with purified water. After that, the coarse emulsions were passed through high-pressure homogenization equipment at 15,000 psi for 8.0 cycles to obtain the final BBR-10-HCPT loaded LM. Finally, the BBR-10-HCPT loaded LM was sterilized by thermal sterilization at 121 °C for 10 min.

### 2.3. Characterization of BBR-10-HCPT-loaded LM

The particle size, particle size distribution and *zeta* potential of BBR-10-HCPT-loaded LM were measured by a Nicomp^TM^ 380 particle sizing system. Entrapment efficiency (EE) was determined by centrifugation using ultrafiltration centrifuge tubes with molecular weight cutoff (MWCO) of 100 kDa. The EE (%) was calculated by the following formula:

$$\text{EE }\left(\%\right)=(C_{\text{total}}V_{\text{total}}-C_{\text{water}}V_{\text{water}})/C_{\text{total}}V_{\text{total}}.$$

*C*_total_: the total concentration of BBR/10-HCPT in BBR-10-HCPT-loaded LM.

*C*_water_: the concentration of BBR/10-HCPT in water phase.

*V*_total_: the total volume of BBR-10-HCPT-loaded LM.

*V*_water_: the volume of water phase.

### 2.4. Determination of BBR and 10-HCPT content

The content of BBR and 10-HCPT in BBR-10-HCPT-loaded LM was determined by HPLC-MS/MS (Li et al., 2014; Li, Yang et al., 2016), the specific operations were as follows: 1.0 mL BBR-10-HCPT-loaded LM was transferred into a 100.0 mL volumetric flask and diluted with methanol. A 2.0 μL aliquot of the solution was injected into the HPLC-MS/MS system for analysis after centrifugation at 15,000 rpm for 5.0 min.

### 2.5. In vitro release of BBR and 10-HCPT

The *in vitro* release behavior of BBR-10-HCPT-loaded LM was evaluated by using the dialysis method. 0.5 mL BBR-10-HCPT-loaded LM was added to the dialysis bag and the molecular weight cutoff was 3500 g/mol. 50.0 mL PBS solution (pH 7.4, 37 °C) containing 0.5% tween 80 and 40% rat plasma were (obtained by dilution with physiological saline) used as dialysis fluid and the vibration speed was 100.0 rpm. 0.5 mL aliquot was withdrawn at 0.5 h, 1.0 h, 2.0 h, 4.0 h, 6.0 h, 8.0 h, 24.0 h, 48.0 h, and 72.0 h separately, and then 0.5 mL fresh medium was added. The concentration of BBR and 10-HCPT was determined by HPLC-MS/MS.

### 2.6. In vitro stability study

LM is a thermodynamically unstable system, and its physical and chemical stability can be affected by many factors. In this study, the effects of temperature and pH value on the stability of the LM were evaluated systematically in order to screen out the optimal preparation. In addition, the chemical and physical stability of BBR-10-HCPT-loaded LM were assessed at accelerated (25.0 °C for 6.0 months) and long-term storage conditions (6.0 °C for 24.0 months). The content, particle size distribution, pH, EE of BBR-10-HCPT-loaded LM were determined at preselected time intervals after placement respectively.

### 2.7. Cytotoxicity assay

The cytotoxicity of BBR-10-HCPT-loaded LM on Hep-3B cell lines was evaluated by MTT assay. The cells were seeded at 6 × 10^3^/well in 96-well plates under the condition of 37.0 °C and 5.0% CO_2_. The culture medium was removed after 24.0 h and added BBR-10-HCPT-loaded LM, 10-HCPT injection, and BBR injection with different concentrations. The incubation time was 48.0 h and 72.0 h respectively to fully evaluate the influence of culture time. Then, 40.0 μL MTT (0.3 mg/mL) was added to each well and removed the supernatant. The precipitated formazan crystals were dissolved by DMSO solution after 4.0 h. The OD values were determined by using an ELISA reader at wavelength 492 nm.

### 2.8. Animal experiment research of BBR-10-HCPT loaded LM

All animal experiments including pharmacokinetics, tissue distribution, and pharmacodynamics were carried out by relevant guidelines that were developed by the China Council on Animal. In addition, these studies were conducted and monitored in accordance with the ethical and scientific principles required by the Declaration of Helsinki. All the procedures were ethically and scientifically approved by the Ethics committee of China Medical University (No 2018PS206K, 2018-02-28).

#### 2.8.1. Pharmacokinetics of BBR-10-HCPT loaded LM

The pharmacokinetics of BBR-10-HCPT-loaded LM, 10-HCPT injection, and BBR injection in SD rats were evaluated in this study (*n* = 6). Briefly, BBR-10-HCPT-loaded LM, 10-HCPT injection and BBR injection were injected respectively. The dose of BBR and 10-HCPT was 3.0 mg/kg and 1.0 mg/kg respectively. Then, 0.2 mL blood was collected at 5.0 min, 15.0 min, 30.0 min, 60.0 min, 2.0 h, 4.0 h, 6.0 h, 8.0 h, 12.0 h, 24.0 h, 48.0 h, 72.0 h, and 96.0 h, respectively after administration. The plasma concentration of BBR and 10-HCPT was measured using a validated HPLC-MS/MS method. The analytical column was poroshell 120 poroshell-C_18_ column, the mobile phase consisted of acetonitrile and 0.1% formic acid. Here, tetrahydropalmatine was chosen as IS and the concentration was 20.0 ng/mL. The ion ionization of BBR, 10-HCPT, and IS were positive ion ionization. Specific chromatographic and mass spectrometric conditions were shown in Tables 1 and 2. 100.0 μL plasma samples and 20.0 μL IS solution were placed in a 10.0 mL EP tube and allowed to mix for 1.0 min. Then, 4.0 mL of extraction solvent (dichloromethane:diethyl ether = 3:2) was added and mixed for 5.0 min by vortex continually, followed by centrifugation at 5000 rpm for 10.0 min. The supernatant was then transferred to a clean tube and evaporated to dryness under nitrogen at 40.0 °C. The residue was reconstituted with 300.0 μL of methanol. After centrifugation at 15,000 rpm for 10 min, a 2.0 μL aliquot of the solution was injected into the HPLC-MS/MS system for analysis.

**Table 1.** Gradient condition of HPLC.

Time (min)	Flow rate (mL/min)	A (%)^a^	B (%)^b^
Initial	0.60	25	75
0.2	0.60	25	75
0.4	0.60	40	60
0.9	0.60	85	15
2.5	0.60	85	15
2.6	0.60	25	75
3.0	0.60	25	75

^a^Acetonitrile; ^b^0.1% formic acid water.

**Table 2.** MS parameters of BBR, 10-HCPT, and IS.

Molecule	Transition	DP (s)	IV (V)	CE (eV)
BBR	336.0→319.8	50	4000	34
10-HCPT	365.3→321.1	45	4500	40
IS	355.9→192.0	35	4500	23

#### 2.8.2. Tissue distribution test of BBR-10-HCPT loaded LM

Tissue distribution of BBR-10-HCPT loaded LM was evaluated in SD rats. BBR injection and 10-HCPT injection were used as a reference. BBR-10-HCPT loaded LM and injections were administrated via the tail vein. The dose of BBR and 10-HCPT was 3.0 mg/kg and 1.0 mg/kg, respectively. Rats were killed at 0.5 h, 1 h, 4 h, 8 h, 24 h, 48 h, 72 h, respectively, post-administration, and then the liver, heart, lung, spleen, kidney, and tumor were collected and weighed. Then, 300.0 μL tissue homogenate and 20.0 μL IS solution were placed in a 3.0 mL EP tube and allowed to mix for 1.0 min. The BBR and 10-HCPT existed in each tissue were extracted with the mixed solvent mentioned before and analyzed by HPLC-MS/MS method.

#### 2.8.3. Determination of HIF-1α

HIF-1α is determined by using a commercial rat enzyme-linked immunosorbent assay (ELISA) kit. Firstly, rat tissue would be cut into pieces, weighed, and homogenized with pH 7.4 PBS for 3 min and then the supernatant was transferred to an EP tube after centrifugation at 2500 rpm for 25 min. Secondly, the calibration solution was prepared by diluting the stock solutions with standard diluent. The calibration concentrations were 5 ng/L, 10 ng/L, 20 ng/L, 40 ng/L, and 80 ng/L. Thirdly, a blank hole without samples and enzyme reagents, standard hole adding 50 μL calibration solution, and test hole adding 40 μL standard diluent and 10 μL tissue sample solution were essential during the test. Then, an enzyme-labeled plate sealed with the membrane was incubated at 37 °C. After 30 min, the solutions were removed, and enough detergent was added to each hole. The detergent was removed and added fresh detergent after 30 s, repeat the above operation 5 times. Fourthly, 50 μL of enzyme-labeled reagent was added to each hole in addition to the blank holes. Similarly, the enzyme-labeled plate was incubated again at 37 °C for 30 min and was washed 5 times with detergent. Finally, color developing reagents A and B were added to each hole in order and finally incubated at 37 °C in dark. After 10 min, 50 μL of termination fluid was added to each hole to terminate the reaction. OD values were measured at a wavelength of 450 nm after setting the blank hole to zero.

#### 2.8.4. In vivo pharmacodynamics evaluations

The anti-tumor effect of BBR-10-HCPT loaded LM was evaluated in nude mice bearing Hep-3B tumor xenograft. The mice were treated with BBR-10-HCPT loaded LM solution, BBR injections, 10-HCPT injection, and saline, respectively. The dose of BBR and 10-HCPT was 3.0 mg/kg and 1.0 mg/kg. Mice were administered preparations twice per week for four weeks. Tumor volume and body weight were measured twice a week. Tumor weight were measured at the end of the experiment. Tumor growth inhibition rate was measured by the following equation:

Tumor inhibition rate (%)=(Tumor weight saline−Tumor weight test group)/Tumor weight saline×100.

## 3. Results and discussion

### 3.1. The appearance and particle size distribution of BBR-10-HCPT loaded LM

**Figure 1.**
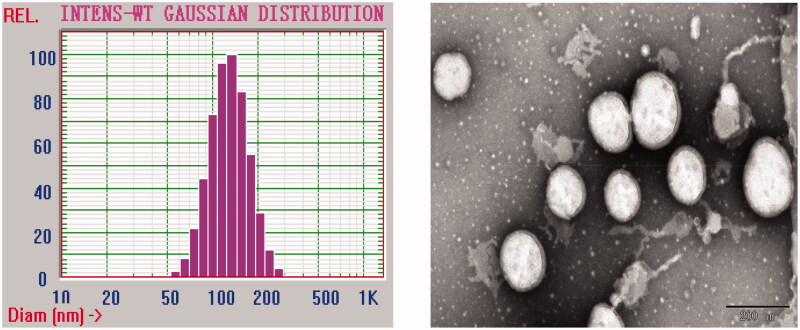
The particle size distribution diagram and transmission electron microscopy of BBR-10-HCPT loaded LM.

Here, the BBR-10-HCPT loaded LM was prepared successfully by applying high-pressure homogenization method. It was found that the prepared BBR-10-HCPT loaded LM was a uniform, yellow, and opaque liquid without flocculation. The particle size distribution was 125.0 ± 21.0 nm and no droplets over 5.0 µm were detected. The particle size distribution diagram and transmission electron microscopy of BBR-10-HCPT loaded LM were shown in Figure 1. The zeta potential was −26 mV due to the presence of phospholipids. It is well known that tumor tissues have specific particle accumulation trends with evident particle size dependence. While BBR-10-HCPT loaded LM possessed proper particle size, so they can accumulate in tumor tissues via the EPR effect. Meanwhile, the blood vessel embolism phenomenon can be avoided because of its small particle size.

### 3.2. Investigation of BBR-10-HCPT loaded LM formulation

#### 3.2.1. Oil phase composition

As is known to all, the composition of the oil phase affects the physicochemical properties of LM because different oils have different characteristics such as solubility, polarity, sterilization stability, etc. In this study, we found that MCT as the oil phase alone could significantly increase the drug loading, but small oil droplets appeared on the surface of the LM after sterilization. In contrast, LCT as the oil phase alone could withstand sterilization, but its drug-loading capacity was insufficient. Meanwhile, the oil phase directly affected the EE of LM. Finally, a mixture of LCT and MCT was chosen as the oil phase. We found that the BBR-10-HCPT loaded LM prepared under the condition of MCT to LCT ratio of 1:1 showed good stability, suitable particle size and narrow particle size distribution, high drug loading, and could withstand thermal sterilization. Therefore, a 1:1 mixture of LCT and MCT was used as the final oil phase, and the oil phase ratio was 10% (w/v).

#### 3.2.2. Emulsifier

The choice of emulsifier was the most critical part in the preparation of LM. The effects of different types of lecithins on the stability of BBR-10-HCPT loaded LM were investigated. It was found that BBR-10-HCPT loaded LM prepared with PL-100M alone could not withstand thermal sterilization. While BBR-10-HCPT loaded LM prepared with S75 could withstand thermal sterilization, but the content of BBR and 10-HCPT decreased significantly, about 5% and 3%, respectively. In contrast, BBR-10-HCPT loaded LM prepared with E80 could withstand thermal sterilization, and the physical and chemical properties had not changed significantly during sterilization, such as the particle size distribution, content, pH value, and *zeta* potential. The potential reasons may be as follows: lecithin is comprised of phosphatidylethanolamine and phosphatidylcholine, but phosphatidylethanolamine possesses a higher emulsifying capacity than phosphatidylcholine. As reported, the content of phosphatidylethanolamine in E80, S75, PL-100M was 9.2%, 9.4%, and 18.0%, respectively. Although PL-100M contains more phosphatidylethanolamine, PL-100M can fully exert its emulsifying capacity only at high pH values between 8 and 10. However, the final pH value of BBR-10-HCPT loaded LM was 5 in this paper. On the one hand, BBR exhibited a pH-dependent solubility with good solubility at a high pH value (>6) and poor solubility at a low pH value (<5). For example, a 5-fold increased solubility of BBR was found when pH increased from 5 to 7. According to the similar miscibility principle, highly soluble drugs would significantly influence the EE of LM. On the other hand, 10-HCPT has a pH-dependent equilibrium between carboxylate form and lactone form under physiological conditions, and the lactone form of 10-HCPT is essential for antitumor activity. Generally speaking, more than 90% of 10-HCPT existed in the form of lactone form at PH5.0. Finally, the pH of the BBR-10-HCPT loaded LM was adjusted to 5.0 in this paper. Therefore, PL-100M was not suitable as an emulsifier for BBR-10-HCPT loaded LM. The decrease in the content of BBR caused by S75 might be related to its low purity. The main component of lecithin was phosphatidylcholine, while the phosphatidylcholine in S75 was the lowest (about 72%), much lower than 83% of E80. Excessive impurities in S75 significantly affected the chemical stability of BBR. Finally, E80 was chosen as the main emulsifier for further study. Additionally, it was necessary to add a co-emulsifier to improve the stability of LM because a small molecule co-emulsifier could increase the stability of the oil–water interface film of LM. Finally, 0.2% pluronic F68 was used as a co-emulsifier.

#### 3.2.3. pH value

pH value also had an important effect on the physicochemical stability of BBR-10-HCPT loaded LM. On the one hand, the emulsifying ability of the lecithin was closely related to the pH of the solution. For example, the emulsifying ability of PL-100M was greatly limited under acidic conditions, which resulting in BBR-10-HCPT loaded LM not being able to tolerate thermal sterilization. Then, the physical stability of the prepared LM during preparation and shelf-life storage could not be ensured. On the other hand, drug solubility, EE, and chemical stability were also highly correlated with pH. Therefore, the appropriate pH was the key to ensure the physical and chemical stability of LM. In this paper, BBR-10-HCPT loaded LM with qualified properties had been obtained at pH 5.0.

#### 3.2.4. Investigation of sterilization conditions

Sterilization was an important measure to ensure the quality of intravenous injections. As we all know, LM was a thermodynamically unstable system, so the choice of sterilization method was extremely important. Among many sterilization methods, thermal sterilization was recognized as the most suitable for LM preparation. The sterilization temperature and time should be systematically investigated after determining the sterilization method. The effect of different time (8.0, 10.0, 12.0, and 15.0) on the properties of LM was investigated at a sterilization temperature of 121.0 °C. It was found that the content of BBR decreased from 0.24% to 0.6% after 12.0 min and 15.0 min of sterilization. The long time of sterilization might destroy the lecithin structure and result in a decrease of emulsifying ability and degradation of drugs. In contrast, the properties of BBR-10-HCPT loaded LM did not change significantly after 10.0 min of sterilization. Finally, the thermal sterilization condition was defined to be 121.0 °C for 10.0 min.

### 3.3. Development of HPLC-MS/MS method for determination of BBR and 10-HCPT

In this paper, an HPLC-MS/MS method had been developed and validated for the determination of BBR and 10-HCPT *in vitro* and *in vivo*. 20.0 ng/mL tetrahydropalmatine was used as IS. The specific HPLC and MS conditions were shown in Tables 1 and 2.

The calibration curves showed good linearity over the concentration range of 0.1 ∼ 100 ng/mL for BBR and 0.5 ∼ 2500 ng/mL for 10-HCPT. The values of accuracy and precision were below 10% for the intra- and inter-day studies, respectively, which may indicate that satisfactory precision and accuracy could be achieved for this assay using the current method validation protocol. The extraction recovery of BBR and 10-HCPT ranged from 97.62% to 99.73%, 94.86% to 101.45% at three concentrations, and LLOQ. The absolute matrix effects were 96.6 3%∼101.82% for BBR and 97.64%∼99.58% for 10-HCPT, which suggested that no ionization enhancement or suppression was observed. In brief, the proposed method can be used to analyze BBR and 10-HCPT.

### 3.4. In vitro drug release of BBR-10-HCPT loaded LM

0.5% tween 80 was added to the release medium to meet the sink condition because BBR and 10-HCPT were slightly soluble substances. 40.0% rat plasma was used as the release medium because rat plasma contained a large number of surface-active substances.

**Figure 2.**
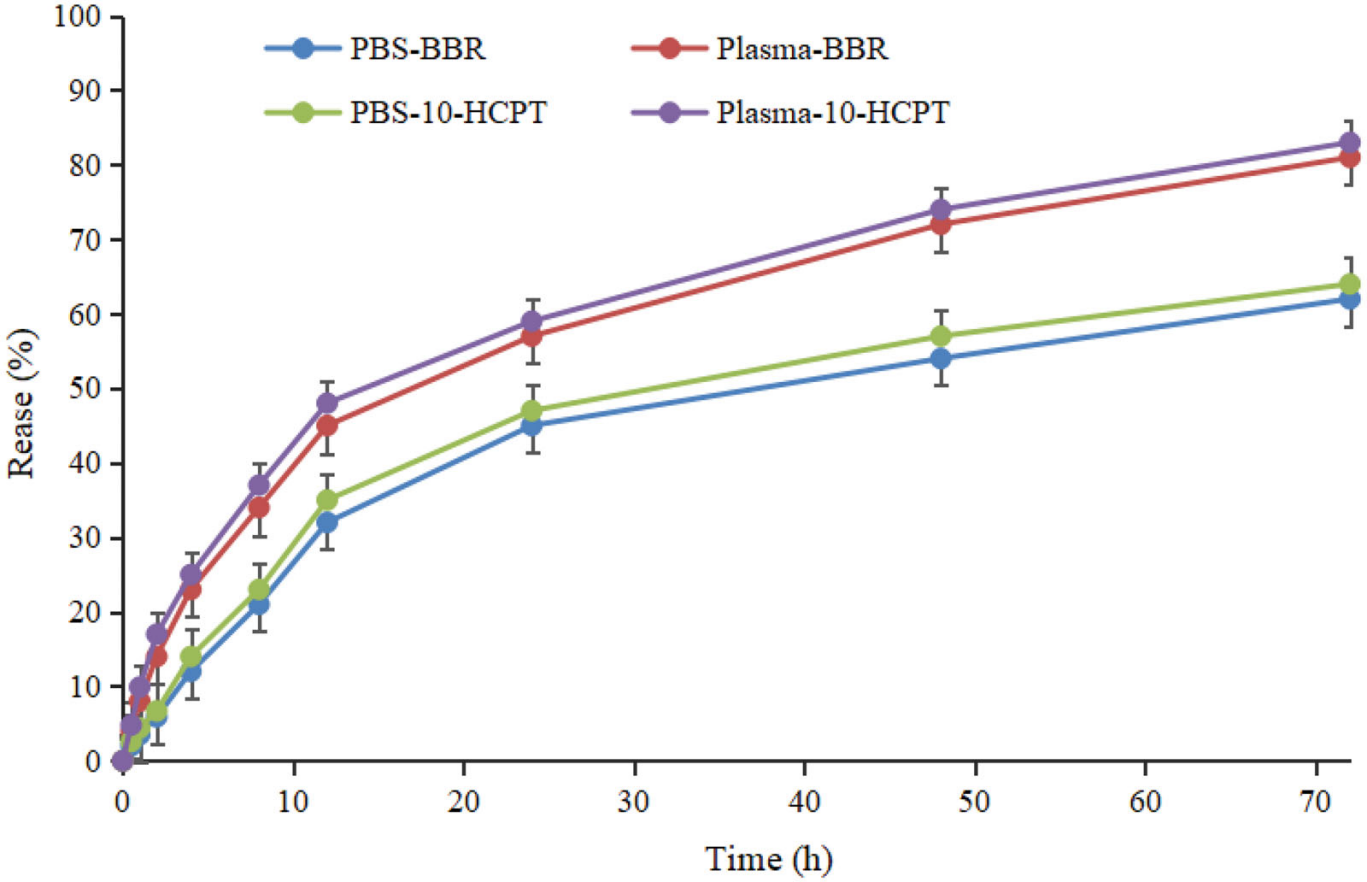
Cumulative release (%) of BBR from LM in PBS and plasma at 37 °C.

Figure 2 showed that the cumulative drug release of BBR and 10-HCPT from BBR-10-HCPT loaded LM was only 3.74% and 4.83% respectively in PBS and 4.24 and 5.75%, respectively in plasma at 1.0 h. The release behavior of BBR and 10-HCPT reached a steady-state until 72.0 h, and the percentage of released BBR and 10-HCPT was 60.4 and 63.6% in PBS and 80.6% and 82.4% in plasma, respectively. It can be seen that BBR-10-HCPT loaded LM can effectively control the release of BBR and 10-HCPT, which showed a significant sustained-release effect in PBS and plasma. The reason may be as follows: firstly, the diffusion of BBR and 10-HCPT form LM was reliant on corrosion and degradation of the carrier because about 90.0% of BBR and 10-HCPT were entrapped in the lipid core and the interface layer of BBR-10-HCPT loaded LM. Secondly, BBR and 10-HCPT had an interaction with MCT, LCT, and lecithin which hindered its diffusion. In contrast, the release of BBR and 10-HCPT in plasma was higher than in PBS, which may be related to the special ingredients contained in the plasma, such as enzymes, surfactants, etc. These enzymes can destroy the structure of LM by hydrolyzing phospholipids, thereby accelerating the release of drugs. To sum up, BBR-10-HCPT loaded LM showed good sustained-release effect and plasma stability *in vitro*, and this result could be employed to predict *in vivo* behavior to some extent.

### 3.5. Stability study

LM is dispersing and thermodynamically unstable systems, comprising mutually immiscible liquids and a surfactant, which has many potential unstable phenomena during storage, such as coalescence, flocculation, phase separation, and degradation of drugs. Therefore, the stability of BBR-10-HCPT loaded LM was assessed in practical conditions to improve security. The results showed that BBR-10-HCPT loaded LM possessed good physical stability at 6.0 °C for 24.0 months and 25.0 °C for 6.0 months respectively. In addition, the content and the EE also did not change significantly during storage. However, the pH of BBR-10-HCPT loaded LM was gradually reduced from 4.93 to 4.79 at 25 °C for 6 months, which might be related to the chemical stability of the oil phase and lecithins. It is known that oil and lecithins are prone to hydrolysis and oxidation because their structures contain large amounts of extremely active unsaturated fatty acids. While the hydrolysis process generates free fatty acids, which results in the decrease of pH values. As mentioned above, the solubility of BBR was positively related to pH value. The solubility of BBR would reduce with the decrease of pH, which would reduce the diffusion of the drug into the aqueous phase, thereby ensuring the EE of BBR-10-HCPT loaded LM. To sum up, a satisfactory BBR-10-HCPT loaded LM with good stability was prepared in this study, and the stability results were shown in Table 3.

**Table 3.** Stability of BBR-10-HCPT loaded LM under different conditions (*n* = 3).

Conditions	Time (min)	EE%	pH	Particle size (nm)	Content (%)
25 °C	0	99.52 ± 0.33	4.93 ± 0.13	125 ± 21	100.2 ± 0.21
	1		4.91 ± 0.15	128 ± 23	99.9 ± 0.23
	2	99.13 ± 0.38	4.87 ± 0.11	133 ± 25	99.7 ± 0.34
	3		4.85 ± 0.15	135 ± 31	99.5 ± 0.42
	6	99.11 ± 0.27	4.79 ± 0.19	136 ± 29	99.6 ± 0.38
6 °C	0	99.52 ± 0.33	4.93 ± 0.13	125 ± 21	100.2 ± 0.21
	3		4.93 ± 0.14	124 ± 22	100.3 ± 0.26
	6	99.45 ± 0.39	4.91 ± 0.17	127 ± 24	100.0 ± 0.24
	9		4.88 ± 0.11	123 ± 23	99.8 ± 0.39
	12	99.32 ± 0.27	4.89 ± 0.09	129 ± 24	99.6 ± 0.32
	18		4.86 ± 0.10	134 ± 26	99.9 ± 0.38
	24	99.12 ± 0.34	4.88 ± 0.11	132 ± 31	99.5 ± 0.34

### 3.6. Cytotoxicity assay

**Figure 3.**
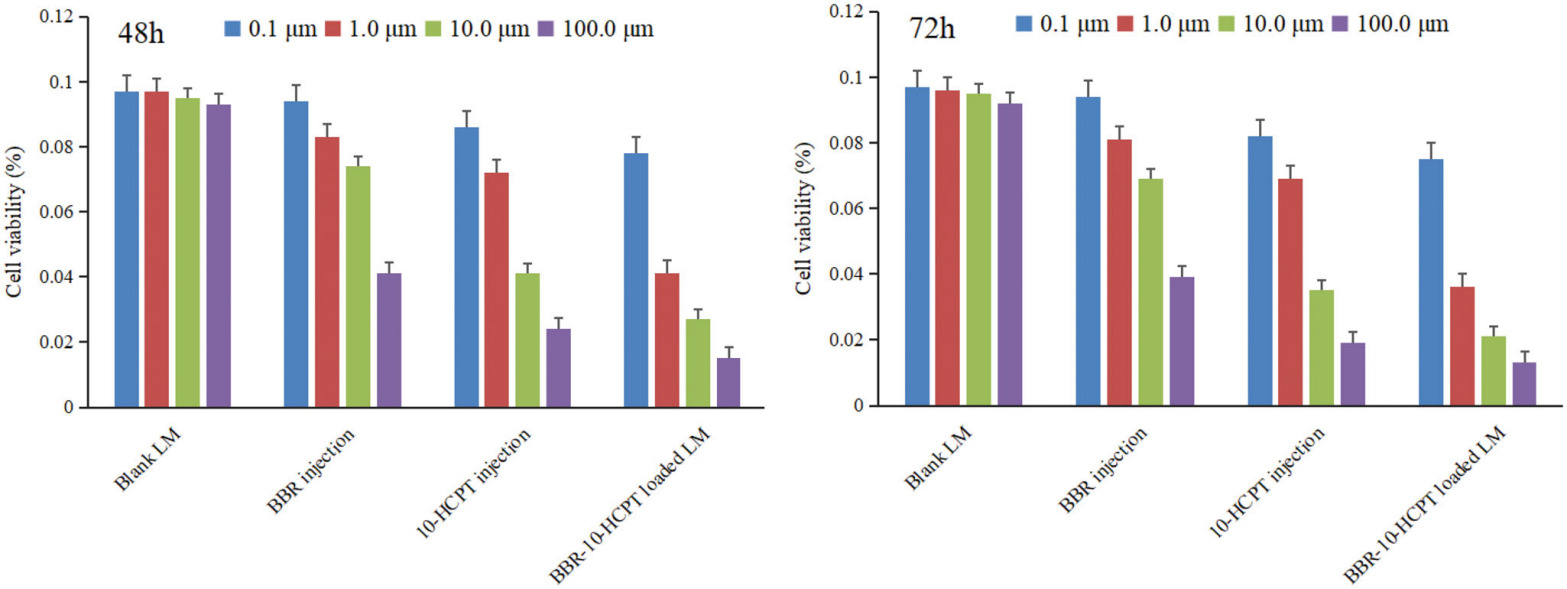
Results of MTT assay on Hep-3B cells after incubation of 48 and 72 h with BBR solutions and BBR-loaded LM at various concentrations.

The cytotoxicity of BBR injection, 10-HCPT injection, and BBR-10-HCPT loaded LM on Hep-3B cells was studied and the results were shown in Figure 3 and Table 4.

**Table 4.** IC_50_ value of Hep-3B cell line cultured with free BBR, free 10-HCPT and BBR-loaded LM for 48 and 72 h.

Incubation time (h)	BBR injection ( µM)	10-HCPT injection ( µM)	BBR-10-HCPT loaded LM ( µM)
48	78.6 ± 12.8	2.05 ± 0.29	0.72 ± 0.18
72	65.3 ± 10.5	1.72 ± 0.18	0.54 ± 0.13

It could be seen that LM was an excellent drug delivery system with high safety and biocompatibility because no obvious inhibition effect on Hep-3B cells by blank LM was found at different concentrations. The BBR-10-HCPT loaded LM demonstrated greater cytotoxic activity than BBR injection and 10-HCPT injection, and this difference was positively correlated with drug concentration and incubation time. The half-maximal inhibitory concentration values for various formulation toward Hep-3B cells were calculated as 78.6 ± 12.8 µM and 65.3 ± 10.5 µM for free BBR (extrapolated), 2.05 ± 0.29 µM and 1.72 ± 0.18 µM for 10-HCPT injection and 0.72 ± 0.18 µM and0.54 ± 0.13 µM for BBR-10-HCPT loaded LM at 48 h and 72 h respectively. The reasons of this phenomenon may be related to the properties of drugs and LM. The uptake of BBR by Hep-3B cells was severely restricted because BBR was an excellent substrate for efflux pump P-glycoprotein (P-gp). Meanwhile, BBR, as an alkaloid, had great polarity, which made it more difficult to enter the cell. So BBR injection showed the highest IC50 among the three preparations. BBR-10-HCPT loaded LM showed lower IC50 than a 10-HCPT injection, which may be related to the characteristics of 10-HCPT. It is well known that 10-HCPT has a pH-dependent equilibrium in the aqueous medium between the lactone and the carboxylate forms. In general, the lactone form is essential for anti-tumor activity and the carboxylate form is almost inactive. While the majority of free 10-HCPT would exist in carboxylate form because the pH of the cell culture solution is about 7.4, which may result in a decrease of cytotoxicity. In addition, the polarity of 10-HCPT would increase when 10-HCPT existed in the form of carboxylate form, which may hinder the access of 10-HCPT into the cell. In contrast, BBR-10-HCPT loaded LM could be easily taken up by endocytosis due to their small size. On the one hand, BBR-loaded LM could effectively shield BBR from P-gp-mediated efflux. On the other hand, BBR-10-HCPT loaded LM could probably enter the cells by different mechanisms such as endocytosis and pinocytosis due to their small size. Meanwhile, nanoparticles of ∼100 nm had been demonstrated to effectively escape *in vivo* nonspecific uptake by reticulo-endothelial system. However, we also found that although BBR-10-HCPT loaded LM showed good cytotoxic activity, the uptake efficiency still needed to be improved. Therefore, additional research was ongoing to increase the uptake of drugs, such as receptor-mediated and targeted modification.

### 3.7. Pharmacokinetics of BBR-10-HCPT loaded LM

**Figure 4.**
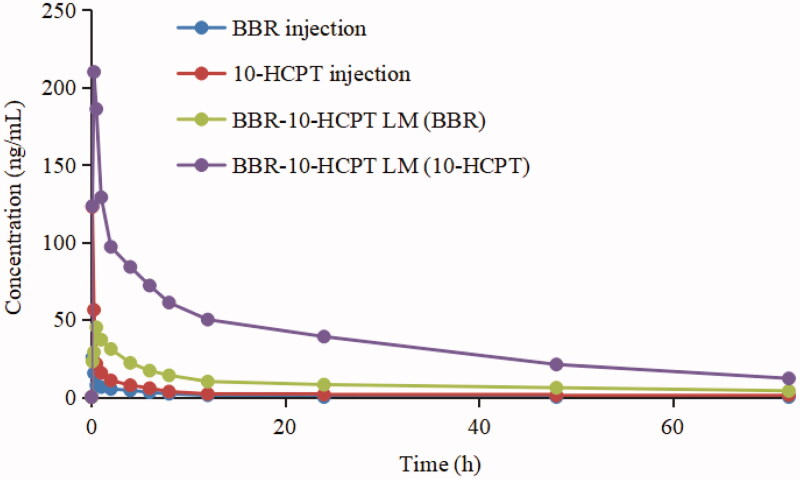
The rat plasma concentration versus time curves of BBR-loaded LM and BBR injection after intravenous administration.

The pharmacokinetics of rats was studied to assess *in vivo* behavior of BBR-10-HCPT loaded LM. The results were shown in Figure 4 and Table 5.

**Table 5.** Pharmacokinetic parameters of BBR and 10-HCPT after intravenous administration of BBR injection, 10-HCPT injection, and BBR-10-HCPT loaded LM (mean ± SD).

Pharmacokinetic parameters	BBR injection	10-HCPT injection	BBR-10-HCPT LM (BBR)	BBR-10-HCPT LM (10-HCPT)
AUC_(0–t)_ (μg/L·h)	56.6 ± 18.4	64.6 ± 10.8	473.5 ± 85.4^a^	1223.4 ± 254.6^b^
AUC_(0–∞)_ (μg/L·h)	57.9 ± 19.6	65.8 ± 11.2	482.8 ± 92.3^a^	1227.2 ± 258.3^b^
CL_z_ (L/h/kg)	54.7 ± 10.7	46.3 ± 9.5	6.6 ± 2.8^a^	5.4 ± 1.1^b^
*C*_max_ (μg/L)	25.6 ± 9.4	124.2 ± 30.1	28.5 ± 7.8^a^	209.4 ± 56.3^b^
*t*_1/2_ (h)	10.5 ± 6.9	0.10 ± 0.02	18.6 ± 3.8^a^	1.9 ± 0.3^b^
*T*_max_ (h)	0.167 ± 0.035	0.083 ± 0	0.50 ± 0.12^a^	0.17 ± 0.08^b^
*V*_d_ (L/h/kg)	123.5 ± 22.4	8.9 ± 2.8	32.5 ± 6.7^a^	0.42 ± 0.05^b^

^a^*p* < .05, compared with BBR solutions; ^b^*p* < .05, compared with 10-HCPT solutions.

It could be seen that BBR injection showed higher apparent distribution volume (*V*_d_), clearance rate (CL), and *C*_max_ than BBR-10-HCPT loaded LM. In other words, BBR could be rapidly and extensively distributed in various tissues after intravenous BBR, which led to some unnecessary side effects such as cardiotoxicity and respiratory inhibition may appear. While 10-HCPT injection could be rapidly eliminated from rat plasma with *t*_1/2_ about 10 min. Meanwhile, 10-HCPT injection group showed low AUC and large CL. In contrast, BBR-10-HCPT loaded LM significantly extended the half-life of BBR and 10-HCPT to 18.6 h and 1.9 h respectively. Furthermore, it was noteworthy that the bioavailability of BBR-10-HCPT loaded LM groups was 8.3-fold and 18.6-fold higher than that of BBR injection and 10-HCPT injection group. The potential reasons of the difference between the three groups might be as follows: (1) BBR-10-HCPT loaded LM was an O/W emulsion with a small particle size, which can avoid the recognition and phagocytosis by the reticuloendothelial system to some extent. So BBR-10-HCPT loaded LM showed long half-life; (2) According to the reports, BBR injection was widely distributed in the body, especially in the lungs, kidney and heart. Then, the relative lack of selectivity toward specific tissue led to the emergence of large Vd and CL, which might also be the source of its serious side effects; (3) BBR-10-HCPT loaded LM had a core-shell structure similar to micelle, so the release of drugs was closely related to the diffusion rate and the structural destruction of LM. Therefore, BBR-10-HCPT loaded LM showed longer *t*_1/2_ than injections, especially for 10-HCPT. In brief, the changes in pharmacokinetic parameters of BBR and 10-HCPT indicated that BBR-10-HCPT loaded LM could change the distribution of BBR and 10-HCPT in different tissues, thus increasing drug selectivity and reducing side effects.

### 3.8. Tissue distribution of BBR-10-HCPT loaded LM

**Figure 5.**
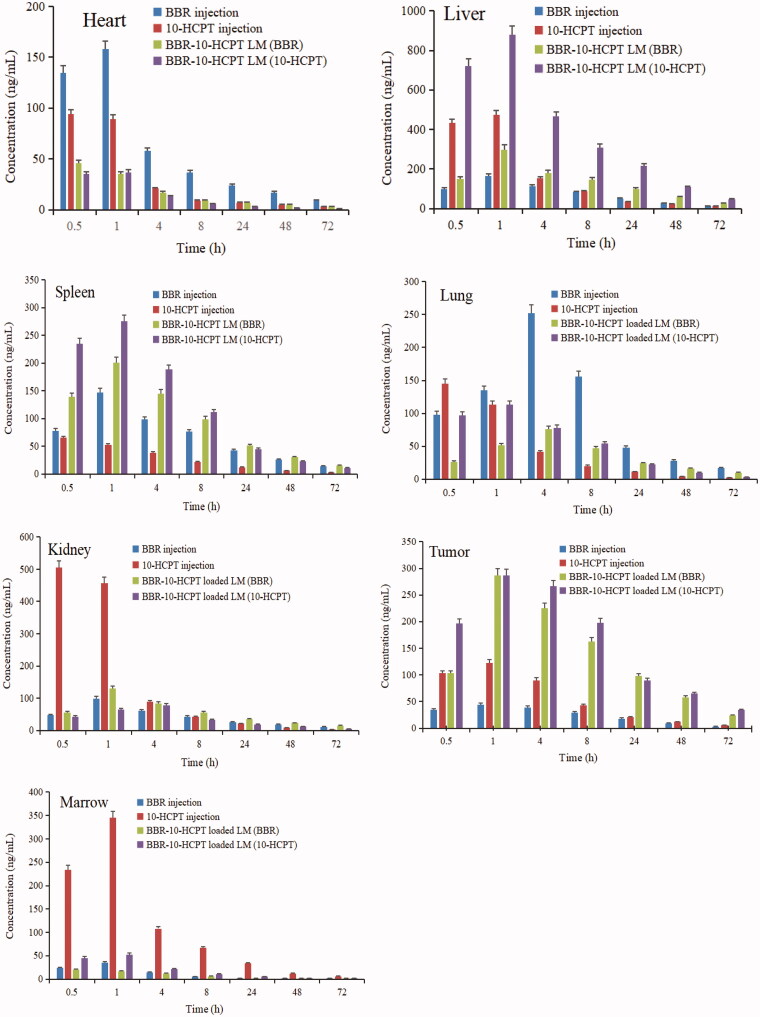
Tissue distribution profiles of BBR after intravenous BBR injection and BBR-loaded LM at the dose of 15 mg/kg.

Tissue distribution of BBR-10-HCPT loaded LM were evaluated and the results were shown in Figure 5.

The results showed that BBR injection was distributed in almost all tissues, especially more in the lung, spleen, kidney, and heart, but less in tumor tissue. As mentioned earlier, BBR injection had serious side effects such as respiratory depression and heart injury in the clinic, which might be directly related to its more distribution in the lung and heart. While 10-HCPT injection was mainly distributed in the heart, kidney and marrow, but it was also less distributed in tumor tissues like BBR. Compared with BBR injection and 10-HCPT injection, BBR-10-HCPT loaded LM significantly changed the tissue distribution behavior of BBR and 10-HCPT. BBR-10-HCPT loaded LM was distributed more in the liver, spleen, and tumors, but less in the lungs and heart, especially in the lung, the concentration was about 30 times lower than that of BBR injection. Furthermore, the distribution *in vivo* verified that BBR-10-HCPT-loaded LM could maintain a longer period of time at a higher level in the liver and tumor compared with injections. The potential reason might be as follows: (1) BBR-10-HCPT loaded LM was a nano-preparation, which was easily phagocytized by the Kupffer cells of the liver and the macrophages of the spleen; (2) BBR-10-HCPT loaded LM could not be trapped by pulmonary capillaries because of small particle size, which resulted in less distribution in the lung; (3) BBR-10-HCPT loaded LM was an O/W type nano-emulsion, which had a long circulation effect in blood and can use the EPR effect of tumor blood vessels to enter tumor tissues. All in all, BBR-10-HCPT loaded LM can improve the selectivity of BBR and 10-HCPT tissue distribution, thereby increasing the efficacy and improving the safety in the clinic.

### 3.9. Determination of HIF-1α

HIF-1α is determined by using a commercial rat enzyme-linked immunosorbent assay (ELISA) kit, and the result was shown in Table 6.

**Table 6.** HIF-1α concentration after intravenous administration of BBR injection, 10-HCPT injection and BBR-10-HCPT loaded LM.

Group	Number of rat	HIF-1α content
Negative	6	347.5 ± 88.4
BBR injection	6	197.3 ± 49.2
10-HCPT injection	6	295.2 ± 78.7
BBR-10-HCPT loaded LM	6	64.4 ± 15.8

It can be seen from Table 6 that BBR-10-HCPT loaded LM showed a higher inhibitory effect on HIF-1α than BBR injection and 10-HCPT injection. The content of HIF-1α in the negative control group, BBR injection group, 10-HCPT injection group, and BBR-10-HCPT loaded LM group was 347.5 ± 88.4 ng/L, 197.3 ± 49.2 ng/L, 295.2 ± 78.7 ng/L, and 64.4 ± 15.8 ng/L respectively. It can be inferred from the results that BBR can effectively inhibit the proliferation of hypoxia-inducible factors in liver cancer tissues, but how to increase the selective distribution of BBR in tumor tissues is the key to its inhibitory effect.

### 3.9. The pharmacodynamics of BBR-10-HCPT loaded LM

**Figure 6.**
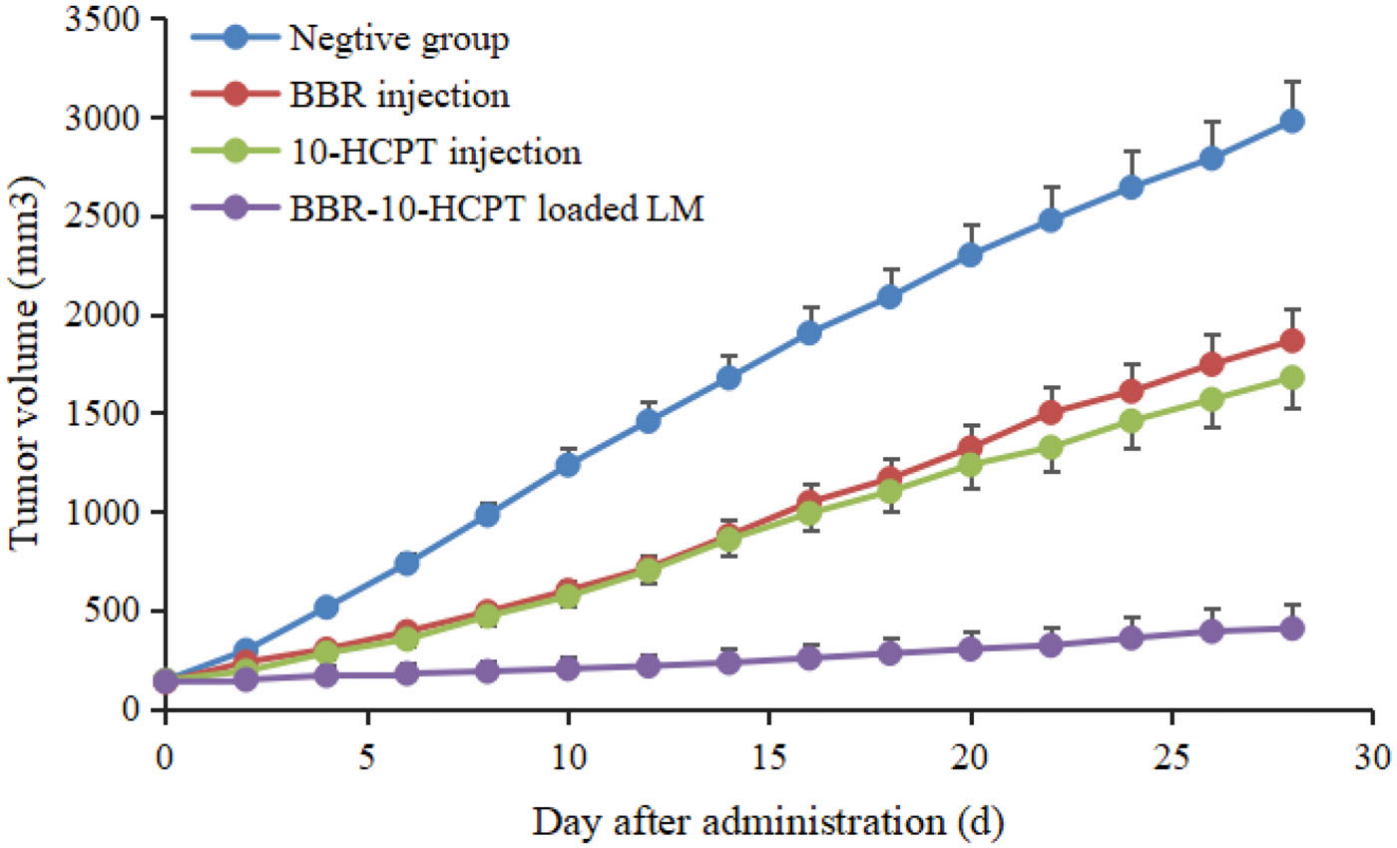
The tumor growth curve of nude mouse after intravenous administration of BBR injection and BBR-loaded LM.

The results of *in vivo* antitumor activity of BBR-10-HCPT loaded LM were shown in Table 7 and Figure 6.

**Table 7.** Anti-tumor effects of BBR injection, 10-HCPT injection, and BBR-10-HCPT loaded LM against Hep-3B cell in nude mouse.

	Dose (mg/kg)	Body weight (g)		Tumor weight (g)	IRT (%)
		Initial	Final		
Normal saline		22.56	32.53	1.48 ± 0.32	
BBR solution	3.0	23.37	34.46	1.02 ± 0.13	31.08
10-HCPT injection	1.0	23.53	32.96	0.92 ± 0.10	37.84
BBR-10-HCPT loaded LM	3.0/1.0	22.89	35.76	0.41 ± 0.15	91.55^a,^^b^

^a^*p* < .05, compared with BBR solutions; ^b^*p* < .05, compared with BBR solutions.

Firstly, there was a significant difference in tumor volume among groups. The tumor volume of the negative control group was much larger than that of other groups. The average tumor weight of the negative group, BBR injection group, 10-HCPT injection, and BBR-loaded LM group was 1.48 ± 0.32 g, 1.02 ± 0.13 g 0.92 ± 0.10 g, and 0.125 ± 0.047 g, respectively. Secondly, there was a certain difference in body weight among groups. After the experiment, the average body weight of the negative group, BBR injection group, 10-HCPT injection group, and BBR-10-HCPT loaded LM group was 32.53 ± 2.43 g, 34.46 ± 2.55 g, 32.96 ± 2.89 g, and 35.76 ± 2.78 g respectively. Thirdly, the daily general physical condition and mental state of each mouse were different. Mice in the control group were mostly in a state of lack of autonomous activity, inactive eating, and weight loss, especially in the late stage of administration. Animals in the BBR injection group showed mild diarrhea, respiratory depression, mild mania, and lack of appetite. Similarly, the mice treated with 10-HCPT injection showed significant pathological state, comprising body weight loss, lackluster hair, and inactive mental mode. In contrast, the mice in the BBR-10-HCPT loaded LM group exhibited a more normal state. The potential reasons might be as follows: (1) BBR-10-HCPT-loaded LM could enter tumor tissues by using the EPR effect of tumor vessels. While BBR injection and 10-HCPT injection lacked the ability to passively target tumor tissues, so the anti-tumor effect of BBR and 10-HCPT could not be fully exerted. Additionally, cardiopulmonary side effects of BBR injection and marrow toxicity of 10-HCPT injection might also affect the anti-tumor effect of BBR and 10-HCPT; (2) BBR-10-HCPT-loaded LM showed a higher inhibitory effect on HIF-1α than BBR injection and 10-HCPT injection. The decrease of HIF-1α can not only directly inhibit the proliferation of tumor cells and angiogenesis, but also indirectly reduce the generation of 10-HCPT drug resistance and the increase of efficacy; (3) BBR-10-HCPT-loaded LM was a nano-preparation with good biocompatibility and little irritation because the excipients used were mostly endogenous substances, so the animals in the test group showed higher tolerance than the other two groups; (4) According to the results of tissue distribution, BBR injections, and 10-HCPT injection were mainly distributed in the lungs, liver, spleen, heart, marrow etc., and can easily induce serious side effects. In brief, BBR-10-HCPT-loaded LM showed a good effect of inhibiting the progression of liver cancer and had great potential research value.

## 4. Conclusions

A novel BBR-10-HCPT-loaded LM was fabricated to treat liver cancer through the synergistic action of 10-HCPT and BBR in this paper. The results showed that the physicochemical stability of BBR-10-HCPT loaded LM was related to many factors, such as the type of emulsifier, pH value, temperature, high-pressure homogenization conditions, sterilization conditions. The *in vitro* release results exhibited that BBR-10-HCPT loaded LM had a significant sustained-release effect in PBS solution and plasma. Meanwhile, it could be seen from the pharmacokinetics and tissue distribution results that BBR-10-HCPT loaded LM could extend the half-life of BBR and 10-HCPT, increase the selectivity of tissue distribution, and improve the bioavailability compared with BBR and 10-HCPT injection. In addition, BBR-10-HCPT loaded LM showed good antitumor activity *in vivo* in nude models bearing Hep-3B cells because of the selective distribution of BBR and 10-HCPT in tumor tissues. To sum up, the BBR-10-HCPT loaded LM prepared by us can significantly improve the efficacy of anti-tumor and reduce its serious side effects, which shows potential research value and broad application prospects.

## Authors’ contributions

All authors made substantial contributions to conception and design, acquisition of data, or analysis and interpretation of data; took part in drafting the article or revising it critically for important intellectual content; gave final approval of the version to be published; and agree to be accountable for all aspects of the work.
